# Identification of Natural Bispecific Antibodies against Cyclic Citrullinated Peptide and Immunoglobulin G in Rheumatoid Arthritis

**DOI:** 10.1371/journal.pone.0016527

**Published:** 2011-01-27

**Authors:** Wei Wang, Jinming Li

**Affiliations:** 1 Graduate School, Peking Union Medical College, Chinese Academy of Medical Sciences, Beijing, People's Republic of China; 2 National Center for Clinical Laboratories, Beijing Hospital, Beijing, People's Republic of China; Universidade de Sao Paulo, Brazil

## Abstract

**Background:**

Previous studies indicate that natural bispecific antibodies can be readily produced *in vivo* when the body is simultaneously stimulated with 2 distinct antigens. Patients with rheumatoid arthritis (RA) usually exhibit persistent immune responses to various autoantigens, raising the possibility that natural bispecific antibodies against 2 distinct autoantigens might exist.

**Methodology/Principal Findings:**

We identified the presence of natural bispecific antibodies against cyclic citrullinated peptide (CCP) and immunoglobulin G (IgG) in RA patients' sera by means of a double-antigen sandwich enzyme-linked immunosorbent assay (ELISA). The spontaneous emergence of bispecific antibodies was confirmed by mixing different proportions of 1 anti-CCP-positive serum and 1 rheumatoid factor (RF)-positive serum *in vitro*. Among the tested samples, positive correlations were found between the presence of bispecific antibodies and both IgG4 anti-CCP antibodies and IgG4 RF (*r* = 0.507, *p*<0.001 and *r* = 0.249, *p* = 0.044, respectively), suggesting that the IgG4 subclass is associated with this phenomenon. Furthermore, bispecific antibodies were selectively generated when several anti-CCP- and RF-positive sera were mixed pairwise, indicating that factors other than the monospecific antibody titers may also contribute to the production of the natural bispecific antibodies.

**Conclusions/Significance:**

We successfully identified the presence of natural bispecific antibodies. Our results suggest that these antibodies originate from anti-CCP and RF in the sera of RA patients. The natural occurrence of bispecific antibodies in human diseases may provide new insights for a better understanding of the diseases. Further investigations are needed to elucidate their precise generation mechanisms and explore their clinical significance in disease development and progression in a larger study population.

## Introduction

We have previously reported the production of natural bispecific antibodies in rabbits that were simultaneously immunized with 2 unrelated antigens [1]. Rabbits immunized with an antigen mixture containing 2 conjugated carrier-hapten(s) (BSA-digoxin and KLH-DNP) produce as many as 6 types of natural bispecific antibodies against antigen pairs that combine randomly [1]. Thus, 2 distinct antigens' simultaneous stimulation is critical for the natural occurrence of bispecific antibodies in vivo. Another research group working on natural bispecific antibodies originating from half-molecule exchanges of IgG4 obtained similar results. Schuurman et al. [2] found that a class of natural bispecific antibodies could be obtained from allergic patients receiving therapeutic injections with 2 different allergens during specific immunotherapy. The mechanism for the production of natural bispecific antibodies is indicated by exchanges of IgG4 half-molecule(s) (a heavy chain and an attached light chain) between 2 antibody molecules [3]–[5]. Taken together, the simultaneous stimulation with 2 distinct antigens is the precondition for the production of natural bispecific antibodies in vivo. Two monospecific antibody populations that are subsequently generated and secreted by the plasma cells interact with each other via a half-molecule exchange, resulting in a new group of antibody molecules exhibiting 2 distinct antigen-binding sites.

Most autoimmune diseases are characterized by the production of various autoantibodies against autoantigens. Rheumatoid arthritis (RA) is a common systemic autoimmune disease of unknown etiology that is characterized by chronically inflamed synovial joints and subsequent destruction of cartilage and bones. The inflammatory synovium is a good place for accommodating targets for a broad range of autoantibodies. The extended disease duration accompanied by persistent immune responses to autoantigens is a situation that is quite similar to our established animal model for natural bispecific antibody production. The main difference is that a spontaneous autoimmune response underlies the disease situation, while active immunization is the driver in the animal model. In another words, RA theoretically possesses the precondition for natural bispecific antibody production, which leads us to speculate that some natural bispecific antibody against 2 distinct autoantigens might exist. Rheumatoid factors (RF) and anti-cyclic citrullinated peptide antibodies (anti-CCP) are frequently found in the sera and synovial fluids of most RA patients and are believed to be pivotal markers of the disease [6]. Furthermore, a positive correlation between anti-CCP and RF is reported in several studies [7]–[9]. On the other hand, studies on subclass distribution indicate that IgG4 is conspicuously elevated in IgG RF and IgG anti-CCP, only secondarily to IgG1 [10]–[12]. Thus, they are more likely to fit the conditions for natural bispecific antibody production such as simultaneous autoantigen stimulation and post-secretion encounters for half-molecule exchanges.

In the present study, we attempt to identify a type of natural bispecific antibodies originating from anti-CCP and RF in RA.

## Materials and Methods

### Patients and controls

Serum samples were obtained from 66 patients who fulfilled the American College of Rheumatology criteria for RA [13]. For comparison, 112 control subjects were tested as well; 62 had the following other autoimmune diseases: 37 with systemic lupus erythematosus (SLE), 16 with primary Sjögren syndrome (pSS), and 9 with scleroderma. Fifty healthy subjects were also included. Serum samples were stored at −80°C until analysis. The RA group consisted of 50 women and 16 men. The mean (SD) age of this group was 48.6 (13.9) years (range: 18–79 years). There was no significant difference between the test and control groups with respect to sex or age.

All subjects included in the study were informed of the nature of the project, and informed verbal consent was obtained before their participation in the study, which was recorded by the physician who explained the study procedure. Written informed consent was not used because of the nature of the study design, which utilized serum samples taken after routine tests. The study protocol and the form of consent were approved by the Ethics Committee of National Center for Clinical Laboratories.

### Bispecific antibody ELISA

Rabbit IgG (Sigma-Aldrich, St. Louis, MO, USA) was labeled with horseradish peroxidase (HRP; Sigma-Aldrich, St. Louis, MO, USA), using the periodate oxidation method as described previously [14]. IgG-HRP conjugates were separated on a SephacrylTM S-200 column (1.0×40 cm; Pharmacia, Uppsala, Sweden). Optimal work concentration for the conjugates was determined by chessboard titrations by using an RF-positive serum pool.

A sandwich ELISA was established with CCP-coated ELISA plates (Immunoscan CCPlus; Euro-Diagnostica, Malmo, Sweden) to detect bispecific antibodies originating from anti-CCP and RF. Before the test, sera were treated with concanavalin to eliminate interference from IgM RF [15]. For the assay, 100 µL serum samples diluted 1∶50 in phosphate-buffered saline (PBS)–1% (m/v) gelatin (G9382; Sigma-Aldrich, St. Louis, MO, USA) was added in duplicate to plate wells. The plates were subsequently incubated for 1 h at room temperature (25°C), followed by 3 washes with PBS–0.05% Tween 20 (PBST). Rabbit IgG-HRP conjugate (100 µL) diluted 1∶400 in PBST-1% gelatin was added to each test well. After 1 h incubation at room temperature, the plates were washed 5 times with PBST. After the addition of 100 µL/well tetramethyl benzidine (T0440; Sigma-Aldrich, St. Louis, MO, USA), the plates were incubated for 30 min at room temperature for color development. The reaction was stopped by the addition of 50 µL of 0.5 M sulfuric acid. Optical densities (O.D.) were read at 450 nm with wavelength correction at 620 nm (OD450/620 nm) in a plate reader (Labsystems, Finland).

### IgG anti-CCP and IgG RF detection

Serum antibodies directed to cyclic citrullinated peptide (anti-CCP) were assessed by a commercial ELISA kit (Immunoscan CCPlus; Euro-Diagnostica, Malmo, Sweden) according to the manufacturer's recommendations. All sera were tested in duplicate, and the results (OD450/620 nm) were averaged.

IgG RF was detected as described previously with some modifications [15]–[16]. Before the assay, sera were treated with concanavalin to eliminate interference from IgM RF [15]. In brief, microtiter plates (Nunc Maxisorp, Roskilde, Denmark) were coated with 100 µL rabbit IgG (5 µg/mL) in carbonate buffer (0.05 M, pH 9.6) at 4°C overnight. After wash steps, the wells were blocked and 1∶100 diluted serum was added (100 µL/well). Wells without antigens were set up for each sample to determine nonspecific binding. The plates were incubated at room temperature for 1 h followed by 3 washes with PBST. HRP-conjugated anti-human IgG (1∶10,000 diluted; Sigma-Aldrich, St. Louis, MO, USA) was applied (100 µL/well). The plates were left at room temperature for 1 h. The wells were washed again, and 100 µL substrate solution was added. Color development was stopped after 30 min, and the absorbance was read at 450 nm. All samples were tested in duplicate, and the results were averaged. A positive serum pool was included in each assay to correct interassay variations. The net absorbance for each sample was obtained by subtracting the nonspecific binding derived from the wells without rabbit IgG.

### IgG4 subclass detection

IgG4 anti-CCP were detected by substituting the anti-human IgG enzyme conjugate of the commercial anti-CCP assay with HRP-conjugated mouse monoclonal antibody against human IgG4 (HP6025; GeneTex, Irvine, CA, USA) diluted 1∶25,600 in PBST-1% gelatin.

For the IgG4 RF assay, EIA microplate wells (Costar) were coated overnight at 4°C with 100 µL rabbit IgG at a concentration of 5 µg/mL in 50 mM sodium carbonate buffer (pH 9.6). The plates were then washed 3 times with PBST and blocked with 200 µL/well PBS–1% gelatin for 1 h at 37°C. After washing, 100 µL of each serum previously treated with concanavalin and diluted 1∶50 in blocking buffer was applied to each well. The plates were incubated for 1 h at room temperature. After washing, 100 µL/well HRP-conjugate IgG4-specific mouse monoclonal antibody against human IgG4 diluted 1∶10,000 in PBST–1% gelatin was applied, and the plates were incubated at room temperature for 1 h. The plates were then washed 5 times, and color was developed as described above. All samples were tested in duplicate, and the results were averaged.

### Mixing test

To validate the bispecificity, anti-CCP and RF were mixed in vitro and the bispecificity and anti-CCP levels were determined.

In a preliminary study, 2 serum samples were selected to conduct the mixing test. Serum #32 was obtained from the RA group, which presented predominantly anti-CCP compared to RF or bispecific antibodies. Serum #3 was obtained from a patient with pSS who possessed a high RF level as well as negative anti-CCP and bispecific antibodies. A healthy serum that was negative in all antibody assays was used as a control.

Serum #32 was diluted (1∶25) and mixed with an equal volume of serial diluted serum #3 (1∶25, 1∶50, 1∶100, 1∶200, 1∶400, or 1∶800) or 1∶25 diluted control serum. Each mixed serum (100 µL) was blended thoroughly and added to 4 CCP-coated microwells in parallel: 2 wells for the bispecific antibody assay and 2 for the IgG anti-CCP assay as described above.

Based on the preliminary assay, we expanded our studies to another 3 anti-CCP-positive but bispecific antibody-negative serum samples (#9, #30, and #40; diluted 1∶25 for the test) and 5 RF predominant serum samples (#6, #33, #53, #58, and #61; diluted 1∶25 for the test). Data regarding anti-CCP, RF, and bispecific antibodies for the samples included in the mixing tests are shown in Table 1.

**Table 1:** Data of anti-CCP, RF, and BsAb for samples included in mixing tests.

Serum No.	Anti-CCP (OD450)	RF (OD450)	BsAb (OD450)
#32	1.300	0.332	0.084
#3	0.075	3.522	0.026
#9	1.215	0.956	0.023
#30	0.556	0.912	0.062
#40	0.947	0.999	0.049
#6	0.067	2.532	0.035
#33	0.085	2.397	0.057
#53	0.055	2.977	0.048
#58	0.069	2.846	0.052
#61	0.061	3.363	0.039

OD450: optical densities at 450 nm with wavelength correction at 620 nm; Anti-CCP: antibodies against cyclic citrullinated peptide; RF: rheumatoid factor; BsAb: bispecific antibodies.

### Statistical analysis

Statistical analysis was performed using SPSS 13.0 for Windows. Antibody levels between different groups were compared by the nonparametric Mann–Whitney *U*-test. Spearman's rank correlation was used to assess the relationship of bispecific antibodies with IgG anti-CCP, IgG RF, IgG4 anti-CCP, and IgG4 RF. Two-sided *p*-values less than 0.05 were considered statistically significant.

## Results

### Bispecific antibody detection in study populations

**Figure 1:**
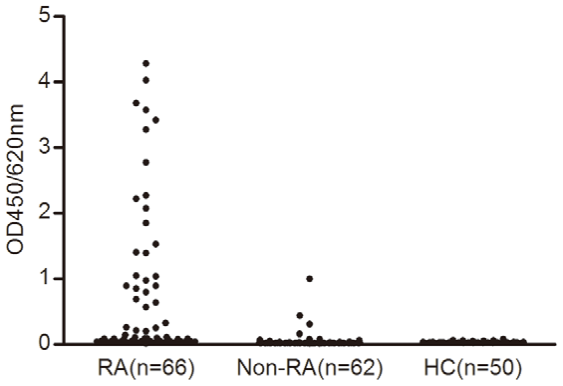
Bispecific antibody results in RA patients and controls. Serum samples from 66 patients with rheumatoid arthritis (RA), 62 patients with other autoimmune diseases (Non-RA), and 50 healthy controls (HC) were analyzed by bispecific antibody ELISA. Results were averaged and are expressed as optical densities at 450 nm (OD450/620 nm).

Patients with RA had higher optical densities (OD450/620 nm) of bispecific antibodies (median OD: 0.096, interquartile range: 0.947) than healthy controls (median OD: 0.030, interquartile range: 0.005) and patients with other autoimmune diseases (median OD: 0.022, interquartile range: 0.012) (*p*<0.001 for both) (Fig. 1).

### Correlations of bispecific antibodies with IgG anti-CCP, IgG RF, IgG4 anti-CCP, and IgG4 RF

**Figure 2:**
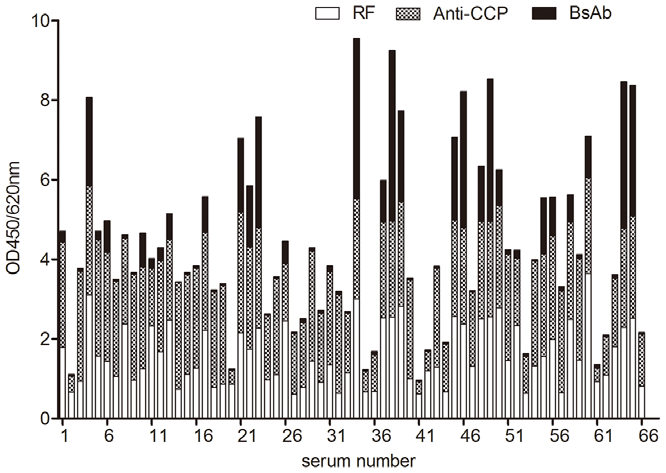
Bispecific antibodies (BsAb), IgG anti-CCP, and IgG RF detection results for 66 RA patients. Numbers along the x-axis are serum numbers. Bars represent the sum of optical densities at 450 nm (OD450/620 nm) for BsAb, IgG anti-CCP, and IgG RF.

**Figure 3:**
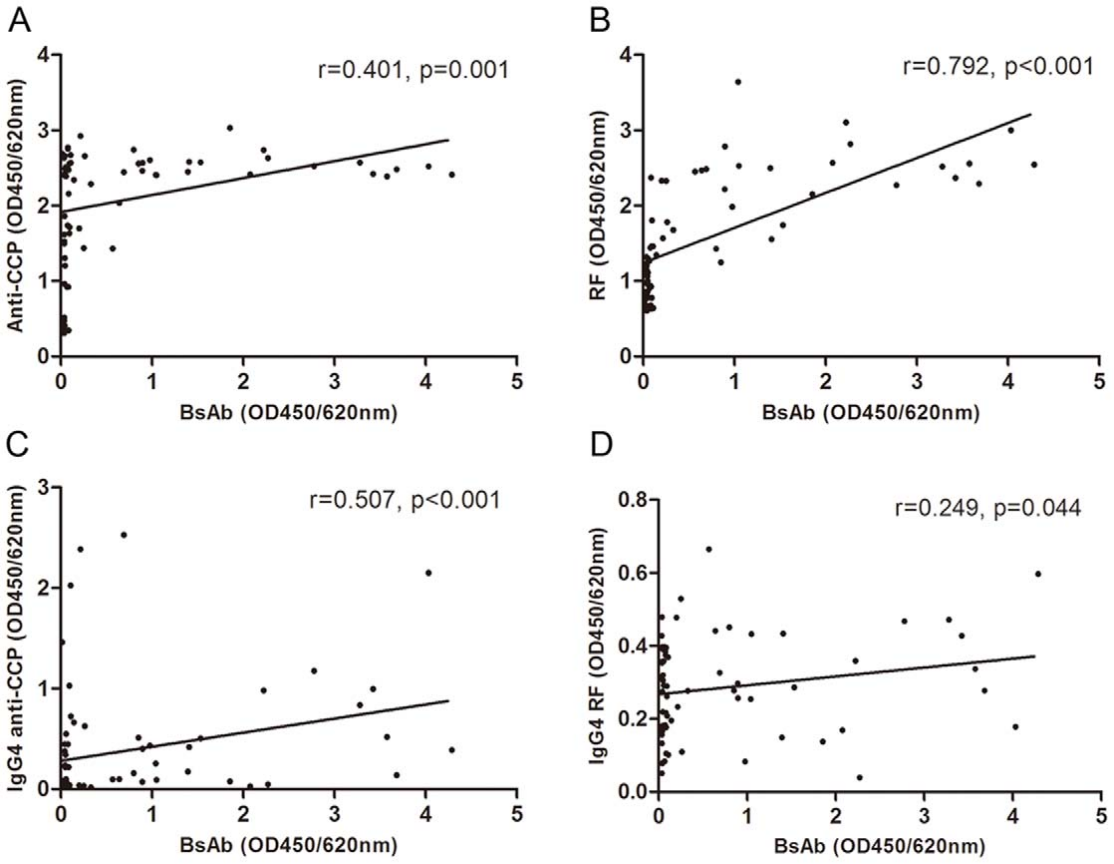
Correlations between bispecific antibodies (BsAb) and various IgGs in RA patients. For the 66 RA patients, BsAb, IgG anti-CCP, IgG RF, IgG4 anti-CCP, and IgG4 RF were determined by ELISA in duplicate. The mean optical densities at 450 nm with wavelength correction at 620 nm (OD450/620 nm) were obtained. The relationship of BsAb with IgG anti-CCP (A), IgG RF (B), IgG4 anti-CCP (C), and IgG4 RF (D) were assessed by Spearman's rank correlation coefficient. Two-sided *p*-values less than 0.05 were considered statistically significant.

The profiles of RF, anti-CCP, and bispecific antibodies for 66 RA patients are shown in Figure 2. The bispecific antibodies were strongly correlated with IgG RF (*r* = 0.792, *p*<0.001) and were reasonably correlated with IgG4 anti-CCP (*r* = 0.507, *p*<0.001); however, relatively weak correlations were found with IgG anti-CCP and IgG4 RF (*r* = 0.401, *p* = 0.001 and *r* = 0.249, *p* = 0.044, respectively) (Fig. 3).

### Mixing test

**Figure 4:**
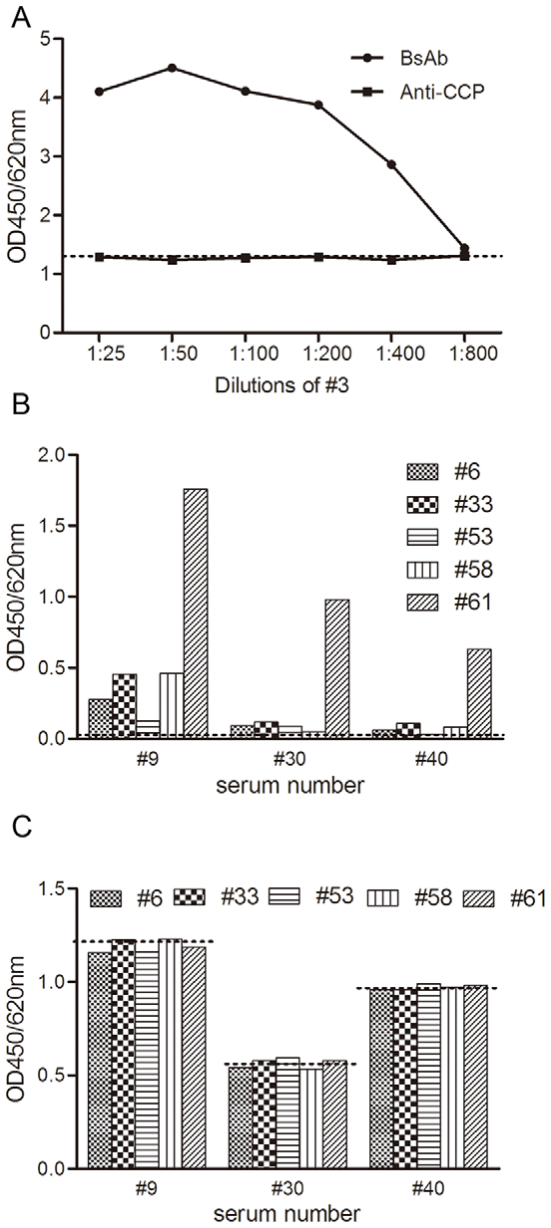
Bispecific antibodies (BsAb) and anti-CCP assay in mixing tests. (A) One anti-CCP-positive but BsAb-negative serum (#32) was mixed with different dilutions of one RF predominant serum (#3); concentrations of BsAb and anti-CCP in the mixtures were determined. Results are expressed as mean optical densities at 450 nm with wavelength correction at 620 nm (OD450/620 nm). Dilutions of #3 are marked on the x-axis. The dashed line indicates the anti-CCP results produced by mixing #32 with normal serum. For (B) and (C), another 3 anti-CCP-positive but BsAb-negative sera (#9, #30, and #40; diluted 1∶25 for the tests) were mixed with 5 RF-containing sera (#6, #33, #53, #58, and #61; diluted 1:25 for the tests) pairwise; BsAb and anti-CCP were detected before and after mixing. The 3 anti-CCP-positive sera are marked on the x-axis. (B) BsAb results before and after mixing. Bars represent mean optical densities in BsAb detection after mixing. The dashed line indicates the mean OD450/620 nm of BsAb for sera #9, #30, and #40 before mixing. (C) Anti-CCP results before and after mixing. Bars represent mean OD450/620 nm in anti-CCP detection after mixing. The dashed lines indicate the OD450/620 nm of anti-CCP for sera #9, #30, and #40 before mixing, respectively.

Mixtures of sera #32 and #3 gave positive results in the bispecific antibody assay, which became weaker with increasing dilutions of #3 (Fig. 4A). As a control, serum #32 was mixed with normal serum and the resultant bispecific signal was extremely low (mean OD: 0.045, data not shown). In parallel, anti-CCP was assessed in mixtures of #32 and #3; the results were nearly the same as those of #32 mixed with normal serum. Likewise, another 3 anti-CCP-positive but bispecific antibody–negative samples were mixed with 5 RF predominant samples pairwise; the bispecific antibody and anti-CCP assays were performed for each pair. The results varied greatly among samples (Fig. 4B). Bispecific antibodies were detected in serum #9 after it was mixed with any of the 5 RF-containing samples; meanwhile, #30 and #40 could only create evident bispecific signals when mixed with #61. Likewise, the anti-CCP results were also the same compared to those before mixing, independent of the bispecific results (Fig. 4C).

## Discussion

In the present study, we report a type of naturally occurring bispecific antibody originating from anti-CCP and RF in RA patients.

The situation described here might be confusing as RF is involved. RF is a common autoantibody directed against the Fc portion of IgG that is either in a complex with its respective antigens or in an aggregated form. RF is an expression product of an individual's immune response to the presence of an aberrant immunoglobulin molecule that is recognized by the body as “nonself.” The majority of RF belongs to the IgM immunoglobulin class, while fewer belong to the IgG and IgA classes [17]. It is widely believed that RF can cause a high level of false positives in indirect ELISA, especially for the detection of IgM antibodies [18]. Thus, it is possible that RF binds to the plate where IgG anti-CCP is captured; thus, what appears to be bispecificity is merely the formation of immune complexes on the plate. To exclude this, 1 anti-CCP-positive but bispecific antibody–negative serum (#32) was mixed with a RF predominant serum (#3) to imitate the status of samples where anti-CCP and RF coexist. If the apparent bispecificity were the results of an artifact due to the formation of a complex of RF with the captured IgG anti-CCP, the RF present in the mix would be expected to interfere with the detection of anti-CCP, thus producing inaccurate results. However, we did not observe any obvious changes when anti-CCP was mixed with different titers of RF (Fig. 4A). This is in agreement with rare reports on the RF interference in anti-CCP tests. Meanwhile, the undisturbed results for anti-CCP after the addition of RF also rule out another potential source of artifacts in the measurement of bispecific antibodies in which RF is complexed with captured anti-CCP via Fc interactions [19]. On the other hand, the fact that not all samples containing higher levels of RF and anti-CCP produce higher bispecific signals also contradicts the artificial suspicion of bispecificity (Fig. 2). Besides, bispecific antibodies could not be detected when mixing #3 with a hepatitis C virus (HCV) antibody–containing serum that was incubated with HCV antigen–coated microwells and subsequently detected by rabbit IgG-HRP (data not shown). Thus, the production of bispecific antibodies is specific between anti-CCP and RF.

Recently, we published a paper describing the establishment of an animal model for the production of natural bispecific antibodies in rabbits. From our results, we draw a conclusion that natural bispecific antibodies are readily produced if a body receives simultaneous stimulation by 2 distinct antigens [1]. We believe this is a general rule for bispecific antibodies that occur naturally in vivo. In humans, a class of natural bispecific antibodies can be obtained from allergic patients receiving therapeutic injections with 2 different allergens during specific immunotherapy, which is in agreement with the results described in our animal models [2]. For RA, the body responds to a variety of autoantigens in conjunction with the development of the disease. This is similar to that described in the animal models, but substituting exogenous antigens with autoantigens. Since RF and anti-CCP are 2 common autoantibodies in RA, there is an increased possibility that their respective autoantigens have more chances to work together. In humans, natural bispecific antibodies are believed to be relevant to the IgG4 subclass, and half-molecule exchanges between 2 IgG4 molecules with distinct specificities are used to explain such natural bispecificity [5]. Although the correlation was not as strong as with IgG RF, the presence of bispecific antibodies in our samples correlated positively with the presence of IgG4 anti-CCP and IgG4 RF (*r* = 0.507, *p*<0.001, and *r* = 0.249, *p* = 0.044, respectively), suggesting that the IgG4 subclass may play a role in the formation of natural bispecific antibodies against CCP and rabbit IgG in RA patients. Previous studies on the subclass distribution of anti-CCP and RF in RA indicate that IgG4 is conspicuously elevated, only secondarily to IgG1; this further confirms the conclusion mentioned above [10]-[12]. Because autoimmune responses arise spontaneously and involve complex phenomena, the occurrence of bispecific antibodies in RA described here can not be simply described by the models of immunotherapy with allergens, in which bispecificity is believed to be solely a function of the IgG4 subclass [2]; other factors related to the disease may play a role.

In our animal model, it is mentioned that the serum environment is critical for conducting half-molecule exchanges in vivo; however, in vitro, some reductive agents such as glutathione (GSH) must be added to facilitate the exchanges [1]. Schuurman et al. [2] demonstrate an in vitro test using 2 IgG4 monoclonal antibodies in the presence of 0.5 mM GSH; we reproduced this using 2 purified monospecific antibodies in the same manner as described in our previous report [1]. However, bispecific antibodies can also be produced in vitro when mixing 2 specific sera in diluent without adding any reductive reagents as described in our mixing tests. A reasonable explanation for this is that the diluted serum matrix may work as accelerators for the production of bispecific antibodies. IgG4 is characterized by the instability of the hinge domains, which is reflected by equilibrium between inter- and intraheavy chain disulfide bonds [20]. Thus, a redox-coupled process might facilitate the half-molecule exchange reaction. It is indicated that the activation, proliferation, and death of cells (apoptosis) are under the control of oxidative balance and are key players in autoimmune pathogenesis and progression [21]. The hallmarks of RA are leukocytic infiltration and activation, synovial proliferation, apoptosis, and the production of a series of autoantibodies. These physiopathological events may in turn affect the redox state of the internal environment. Consequently, we postulate that environmental factors that favor the assembly of bispecific antibodies, such as the oxydo-reductive potentials of the internal environment, might account for the production of bispecific antibodies in our mixing tests. This also appears to be the explanation for the diversity of the bispecificity among different serum pairs in mixing tests. Nevertheless, this speculation needs to be carefully checked out by future research.

The bispecific antibodies that occur naturally in vivo may play a special role in the immune responses associated with human diseases. Natural bispecific antibodies are functionally monovalent and therefore cannot crosslink antigens and trigger pathophysiological effects associated with antigen aggregation. Besides, these antibodies, which are often of the IgG4 type, are unlikely to burden inflammation but rather ease the situation because of their inability to activate complement. Furthermore, the anti-inflammatory activity of bispecific antibodies, which originates from the half-molecule exchanges of 2 IgG4 molecules, was confirmed in vivo in a rhesus monkey model with experimental autoimmune myasthenia gravis [5]. Considering these aspects, we postulate that the production of natural bispecific antibodies originating from anti-CCP and RF may be a beneficial event for RA patients since these antibodies have the potential to serve as indicators for disease remission.

In summary, we identified a type of antibody in RA that exhibits bispecificity against CCP and rabbit IgG. The presence of specific antibodies of the IgG4 subclass, together with yet unsure environmental factors, collectively influence the generation of bispecific antibodies. The natural bispecific antibodies involved in human diseases may have particular significance in the understanding of these diseases from a new perspective. Since natural bispecific antibodies are thought to have anti-inflammatory activities, it would be intriguing to further investigate the clinical relevance of bispecific antibodies for RA patients with respect to disease progression and prognosis, as well as explore other autoimmune disease-associated bispecific antibodies.
